# Reaching Visible Light Photocatalysts with Pt Nanoparticles Supported in TiO_2_-CeO_2_

**DOI:** 10.3390/ma15196784

**Published:** 2022-09-30

**Authors:** Ixchel Alejandra Mejia-Estrella, Alejandro Pérez Larios, Belkis Sulbarán-Rangel, Carlos Alberto Guzmán González

**Affiliations:** 1 Department of Water and Energy, University of Guadalajara, Campus Tonalá, Tonalá 45425, Mexico; 2 Department of Engineering, University of Guadalajara, Campus Altos, Tepatitlán de Morelos 47635, Mexico; 3 Department of Applied Basic Sciences, University of Guadalajara, Campus Tonalá, Tonalá 45425, Mexico

**Keywords:** nanocatalysts, photocatalysts, band gap energy, sol-gel and impregnation method

## Abstract

Nanostructured catalysts of platinum (Pt) supported on commercial TiO_2_, as well as TiO_2_-CeO_2_ (1, 5 and 10 wt% CeO_2_), were synthesized through the Sol-Gel and impregnation method doped to 1 wt% of Platinum, in order to obtain a viable photocatalytic material able to oxidate organic pollutants under the visible light spectrum. The materials were characterized by different spectroscopy and surface techniques such as Specific surface area (BET), X-ray photoelectron spectroscopy (XPS), XRD, and TEM. The results showed an increase in the diameter of the pore as well as the superficial area of the supports as a function of the CeO_2_ content. TEM images showed Pt nanoparticles ranking from 2–7 nm, a decrease in the particle size due to the increase of CeO_2_. The XPS showed oxidized Pt^2+^ and reduced Pt^0^ species; also, the relative abundance of the elements Ce^3+^/Ce^4−^ and Ti^4+^ on the catalysts. Additionally, a shift in the Eg band gap energy (3.02–2.82 eV) was observed by UV–vis, proving the facticity of applying these materials in a photocatalytic reaction using visible light. Finally, all the synthesized materials were tested on their photocatalytic oxidation activity on a herbicide used worldwide; 2,4-Dichlorophenoxyacetic acid, frequently use in the agriculture in the state of Jalisco. The kinetics activity of each material was measured during 6 h of reaction at UV–Vis 190–400 nm, reaching a removal efficiency of 98% of the initial concentration of the pollutant in 6 h, compared to 32% using unmodified TiO_2_ in 6 h.

## 1. Introduction

As the population continues to grow, pollution has increased in all water resources, producing an urgent need to create solutions to remediate it. Studying the literature regarding a reliable technology for water treatment able to oxidate persistent organic molecules heterogeneous catalysts, it has been proven to be able to remove a wide range of contaminants [1,2,3]. By doping the reaction with metallic and nanometric catalysts helps to increase the surface energy of the individual particles, increasing the probability of aggregation, which can reduce the specific surface area of the catalyst and its efficiency since they are widely used materials due to their relatively low cost, and they can be reused [4,5]. To avoid aggregation, it is important to immobilize the active metal nanoparticles on mesoporous solid support, as well as to transform the metal into its oxide form [6].

The morphological structure of the catalyst support determines the dispersion of the nanoparticles and the surface area of the catalytic active sites. Various semiconductor materials including titanium dioxide (TiO_2_), zinc oxide (ZnO), vanadium pentoxide (V_2_O_5_), cerium oxide (CeO_2_) and tungsten trioxide (WO_3_) have been extensively studied by photo catalysis reaction [1,7,8]. One of the most researched compositions for a support system is TiO_2_ because it is an effective, inexpensive, and stable photocatalyst used for the decomposition of organics [1,4]. However, TiO_2_ can only absorb the ultraviolet portion and can only take advantage of about 4% of the intensity of the sunlight spectrum due to its high bandgap (3.0–3.2 eV) [1,9]. This represents a major limitation since its photocatalytic properties are not fully used; even so, an alternative that has been explored to extend its photo response range to the region is to dope the surface with metallic nanoparticles or combine it with another support [4]. Selected methods like doping and composites have been attempted to achieve photo-initiation into the visible spectrum, therefore decreasing cost and increasing efficiency [6]. Another support that has gained importance recently and that can be used as a photocatalyst is CeO_2_. This is due to its unique redox properties that consist of reversibly creating and eliminating oxygen vacancies on the surface [10]. 

The relation between the photocatalytic activity of TiO_2_ and CeO_2_ composite under UV and visible light has been studied [8,11,12,13]. Liu et al. in 2005 found that TiO_2_-CeO_2_ under visible illumination exhibits more photocatalytic activity than pure TiO_2_ and CeO_2_ films. Another study reported by Tian et al. in 2013, who prepared heterostructures of CeO_2_/TiO_2_ nanobelts using a hydrothermal method. These authors found that both UV and visible photocatalytic activities of CeO_2_/TiO_2_ nanobelt heterostructures were enhanced compared to TiO_2_ nanobelts and CeO_2_ nanoparticles. More recently, Henych et al. (2021) found the strong interaction of Ti with Ce within the composites led to the formation of Ce^3+^ and Ti^<4+^ states, reduction of titania crystallite size, change of acid-base and surface properties, and synergetic effects that are all responsible for highly improved degradation efficiency of organophosphorus compounds. In addition, the thermocatalytic, photocatalytic, and photothermocatalytic oxidation of some volatile organic compounds, 2-propanol, ethanol, and toluene, were investigated over brookite TiO_2_-CeO_2_ composites [11]. Other studies have focused on improving catalyst synthesis methods using green methods [14] or adding doping of TiO_2_-CeO_2_ supports to improve photocatalytic properties [15,16]. 

In order to improve the combination of TiO_2_ and CeO_2_ supports, the incorporation of platinum nanoparticles was studied in this research. Platinum nanoparticles are advantageous in biological, biosensor, electro-analytical, analytical, and catalytic applications [17]. They are unique because of their large surface area and their numerous catalytic applications, such as their use as automotive catalytic converters and as petrochemical cracking catalysts [18]. As mentioned above, the present work takes advantage of the combined photoactivity properties of two semiconductors TiO_2_-CeO_2_ supports and the platinum nanoparticles forming Pt/TiO_2_-CeO_2_ photocatalyst. The TiO_2_-CeO_2_ supports were prepared by sol-gel method at different contents of the cerium oxide (2–10 wt%) and the platinum nanoparticles (1.0 wt%) were prepared by the impregnation method to obtain Pt/TiO_2_-CeO_2_ photocatalysts. This project is innovative because it will use the semiconductor, TiO_2_ mixed with CeO_2_, mechanically alloyed, to shift photo-initiation into the visible range.

## 2. Materials and Methods

### 2.1. Materials

Cerium (IV) oxide reagent grade, 97%, was used as a support for the catalyst in the sol-gel method. The TiO_2_ P25 reagent grade 99.5%, commercial salt of hexachloroplatinic for a precursor of nanoparticles of Pt at 37.5% purity (H_2_PtCL_6_ * 6H_2_O), the reactive used to reach pH in the preparation methods was nitric acid (HNO_3_) reagent at 65%, ethanol (C_2_H_5_OH) at 99.8% hydrochloric acid (HCl) at 99.9%. All reagents were obtained from Sigma-Aldrich (Toluca, México).

### 2.2. Support Preparation

#### 2.2.1. Impregnation Synthesis 

The TiO_2_ supports was prepared using TiO_2_ Degussa P25 (Aldrich, 99.5%) it was first placed in a thermal treatment at 500 °C for 2 h with an airflow of 50 mL/min. The CeO_2_ was incorporated into the TiO_2_ with different contents (1, 5, and 10% by weight of CeO_2_) with an aqueous solution of Ce (NO_3_)_3_ * 6H_2_O. This solution was added to the TiO_2_ that was placed in a ball flask. The mixture was stirred for 3 h on a rotary evaporator. Afterwards, the samples were dried under vacuum in a 60 °C water bath. Subsequently they were dried in an oven at 120 °C for 12 h and calcined at 500 °C for 4 h with an air flow (50 mL/min) and a heating ramp of 2 °C/min. This process was performed in duplicate.

#### 2.2.2. Sol-Gel Synthesis 

To prepare the TiO_2_ support material by sol-gel, titanium IV butoxide (Aldrich, 97%) was used as a TiO_2_ precursor with water, ethanol, and a few drops of HNO_3_ to fix the pH in the solution to 3. The preparation of the supports was made in a three-necked flask, mixing dropwise the n-Butoxide into the mix in the water/alkoxide solution (8:1 molar ratio). Then the mixture was placed to reflux and stirred vigorously for 24 h. The temperature of the preparation was maintained in a range between 75–80 °C. The samples were dried under vacuum on a rotary evaporator with a 75 °C water bath. Finally, the supports were calcined at 500 °C for 4 h with an airflow (50 mL/min) and a heating ramp of 2 °C/min [19]. 

The process to add the CeO_2_ into the support web was the same as described previous for the TiO_2_ sol-gel, with the difference of adding the reagent Ce (NO_3_)_3_ * 6H_2_O by previously preparing a solution in order to obtain the desired percentages in the support web. This process was performed in duplicate and in parallel.

### 2.3. Pt Catalysts Preparation

The catalysts were prepared by wet impregnation, using the support material previously synthesized with TiO_2_ and CeO_2_. Prior to catalyst preparation, both TiO_2_ and TiO_2_-CeO_2_ supports were previously air-dried at 100 °C for 24 h. Subsequently, the supports were added to a ball flask to which a 0.001 M hydrochloric acid solution was used to adjust the pH. Then the mixed solution with the support was left stirring until it became homogenized, after which the Pt solution was added, and commercial salt of H_2_PtCl_6_ * 6H_2_O at 37.5% purity was employed as a precursor for Pt in order to obtain a semiconductor material with a 1% weight metal content. The suspended solution was heated to 60 °C with vigorous stirring for 4 h. The leftover solids were dried in the oven at 120 °C for 24 h and calcined at 500 °C with an airflow of 1 cm^3^/s and heating rate of 2 °C/min. Finally, the samples were placed in a vacuum desiccator in amber glass vials wrapped in foil paper to mitigate the exposure to light [20].

### 2.4. Characterization Techniques

The TiO_2_-CeO_2_ supports and the platinum nanoparticles that form the Pt/TiO_2_-CeO_2_ photocatalyst synthesized by sol-gel and impregnation at different concentrations were characterized to determine which method improved their photocatalytic properties. The determination of the specific surface area was carried out using the standard Brunauer, Emmett and Teller (BET) method using nitrogen physisorption in Micromeritics ASAP 2020. The X-ray diffraction (XRD) was used to determine their phases and crystallinity. This was carried out using an Empyrean by Malvern Panalytical, Almelo, equipped with Cu-Kα radiation (λ = 0.154 nm). The phase content of anatase and rutile were calculated with the XRD intensity of the characteristic peaks of the phases [21,22], as shown in Equation (1).

(1) $$W_{A}=\frac{K_{A}I_{A}}{\left(K_{A}I_{A}+I_{R}\right)}$$

where W_A_ is the mole fractions of anatase, I_A_ and I_R_ are the X-ray integrated intensities of the anatase and the rutile, respectively, and K_A_ = 0.886.

The presence of elements in the catalyst and the percentage of each element were determined using X-ray photoelectron spectrometry (XPS) Phoipos 150 (ESCALAB 210, VG Scientific Ltd., East Grinstead, UK) and Raman spectroscopy (Cora 5500 Anton Paar, Anton Paar, Germany). Transmission Electron Microscopy (TEM) has been used to explicate the innermost structure, morphology, and exact particle size of the composite system (FEI TITAN G2 80–300, Hillsborough, OR, USA) operated at 300 keV. The UV–Vis (UV–Visible) spectrophotometer (Shimadzu UV-2600, Kyoto, Japan) was used to determine the energy level of the band gap for all composites of Pt/TiO_2_-CeO_2_ photocatalyst synthesized.

### 2.5. Photocatalytic Reaction

The photodegradation experiments of 2,4-D was carried out at room temperature, using a slurry reactor, a glass beaker of 200 mL capacity with 150 mL of a mother solution with 200 ppm of 2,4-Dimethylamine salt, and 200 mg of catalyst mixed with air (BOYUS air pump 4000 B, with pressure of 0.012 MPa and an output of 3.2 L per minute). The reaction was kept in agitation for 30 min in complete darkness until the adsorption desorption equilibrium had reached light striking the reactor with a UV lamp (4 watts). The run time for adsorption tests was 6 h under darkness, at the natural pH of the slurry. An example was obtained every 30 min, using a filtrating syringe to extract 4 mL of the slurry and using a membrane to separate the suspension material. Every example was measured in a UV–Vis (UV-2600 Shimadzu) at 190–400 nm. To obtain the kinetics activity of each material. The concentration of the reaction was calculated from the absorption band at 282 nm, applying the equation of Beer–Lambert. The conversion percentage was calculated using the Equation (2).

(2) $$X_{2,4D}=\frac{2,4D_{0}-2,4D_{f}}{2,4D_{0}}\times 100\%$$

where X_2,4D_ is the percentage of the 2,4-D conversion, 2,4D_0_ is the concentration of the pollutant at the beginning of the reaction, and 2,4D_f_ is the concentration of the pollutant at the end of the reaction.

The heterogenous photocatalysts were carried out by employing the following catalyst: Aeroxide P-25^®^ Commercial TiO_2_, TiO_2_-CeO_2_ (5, 10 wt%), Pt-TiO_2_-CeO_2_ (5, 10 wt%) synthesis by impregnation and TiO_2_-CeO_2_ (1, 5, 10 wt%), Pt-TiO_2_-CeO_2_ (1, 5, 10 wt%) synthesis by sol-gel.

## 3. Results and Discussion

### 3.1. Specific Area by the BET Method

In order to investigate the effect that was created in the surface of the support with the addition of CeO_2_ in different concentrations, several material characterization techniques were made. Starting with the specific area determined by the BET method, as well as the average pore diameter of the TiO_2_ and TiO_2_-CeO_2_ supports, we can observe that the effect of adding CeO_2_ to TiO_2_ increased the pore diameter and the specific area decreased. The impregnation method generated a higher diameter of pores and low specific surface area than the sol-gel method, probably because the sol-gel method had better dispersion of CeO_2_ in the TiO_2_. Similar effects have been reported by other authors [23]. Table 1 shows the results of the specific surface area, and the pore diameter of the catalyst support.

### 3.2. X-ray Diffraction (XRD)

**Figure 1:**
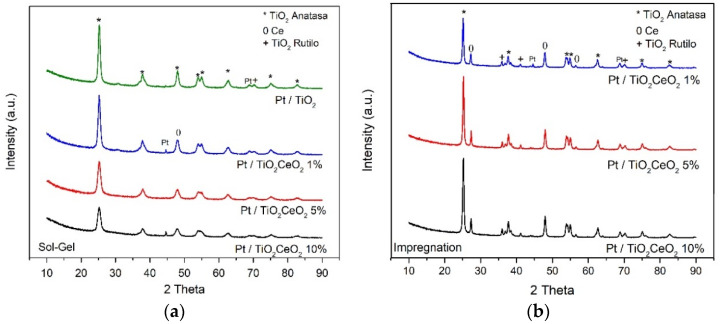
XRD of the catalysts Pt/TiO_2_ and Pt/TiO_2_-CeO_2_: (**a**) Sol-gel and (**b**) Impregnation.

An X-ray diffraction characterization test was performed to evaluate the content of the anatase and rutile phases in the supports for both methods. The crystalline phases of TiO_2_ and TiO_2_-CeO_2_ can be seen in the diffraction patterns found in Figure 1. For the Pt-TiO_2_ catalyst and the mixed oxides TiO_2_-CeO_2_ synthesized by sol-gel and impregnation, the presence of the peaks 2ϴ = 25.19, 37.60, 53.95, 54.36, 62.68, 75.04, and 82.7 are attributed to the anatase phase, corresponding to the plane (JCPDS no. 21-1272). Sol-gel presented the rutile phase whose peaks associated with the phase are 2ϴ = 70.16 and impregnation in 2ϴ = 35.81, 41.04, and 70, according to JCPDS with reference number 23-0278. The anatase phase is dominant in both methods due to the heat treatment to which the material was subjected. This is good since the anatase phase is a better photocatalyst than rutile because the exciton diffusion is twice as long [6]. In addition, in the sol-gel method it is possible to observe a decrease in the peaks of the anatase phase due to the increase of CeO_2_ in the network of the support. For CeO_2_ the peaks 2ϴ = 47.80, are associated with the cerenite phase that corresponds to a cubic packing of CeO_2_. With the impregnation method, CeO_2_ was observed at 2ϴ = 27.29, 47.80, and 56.54. In Pt/TiO_2_-CeO_2_ catalysts, the cerenite phase was observed very little, probably because it is very dispersed within the TiO_2_ structure, this can be observed in the shift to the right of the characteristic peaks of the TiO_2_ (JCPDS, no. 04-0802).

### 3.3. Raman Spectroscopy

**Figure 2:**
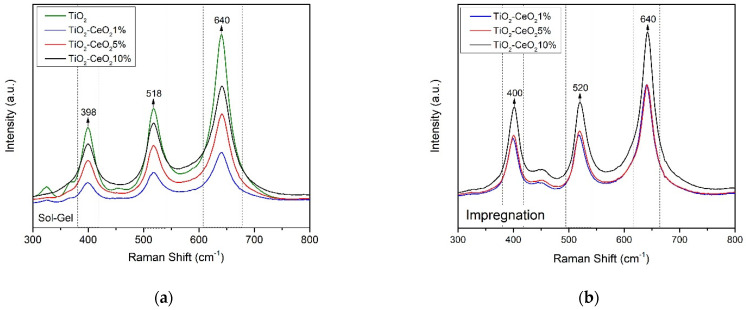
Raman spectrum for the support system TiO_2_, TiO_2_-CeO_2_ (1, 5, 10 wt%): (**a**) Sol-gel and (**b**) Impregnation.

On the other hand, as seen in Figure 2, in the Raman spectra of the TiO_2_ supports and the mixed oxides TiO_2_-CeO_2_, which were prepared by the sol-gel and impregnation method, peaks corresponding to the anatase phase 398–400, 518–520, and 640 cm^−1^ are observed. Observing a slight Raman shift, which means that CeO_2_ has been integrated into the structure of the TiO_2_ support. It is assumed that the ≡ Ti-O-Ti ≡ bonds of the corresponding TiO_2_ network of the anatase phase are disturbed by the presence of cerium oxide, which suggests some substitutions of the Ti^4+^ by Ce^4+^ that form ≡ Ti-O-Ce ≡ bonds in the structure of titanium oxide.

### 3.4. Transmission Electron Microscope (TEM)

**Figure 3:**
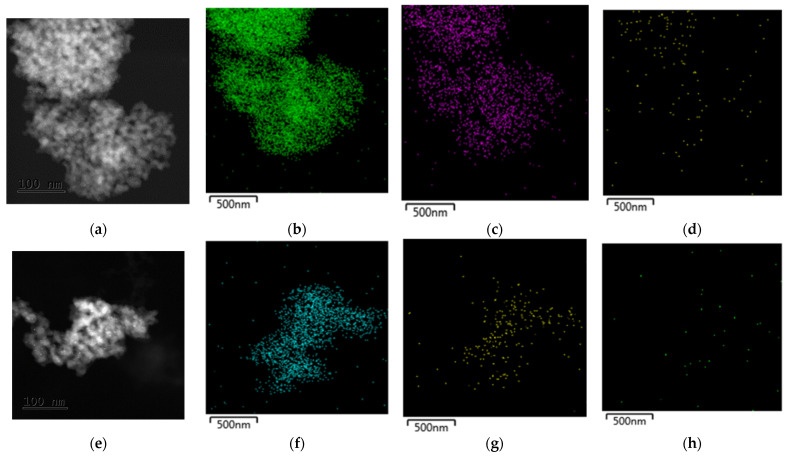
Transmission electron microscopy images of the Pt/TiO_2_-CeO_2_ 10%: (**a**) Sol-gel to 100 nm scale, (**b**) Sol-gel take α of the Ti, (**c**) Sol-Gel take α of the Ce, (**d**) Sol-Gel take α of the Pt, (**e**) Impregnation to 100 nm scale, (**f**) Impregnation take α of the Ti, (**g**) Impregnation take α of the Ce, and (**h**) Impregnation take α of the Pt.

In the transmission electron microscope (TEM) information obtained is the particle size by analyzing the images in software capable of measuring the diameter of the particle on a nanometric scale. Their respective alpha images of each of the elements and how it is dispersed inside and outside the support is shown in Figure 3. The particle size dispersion histograms were obtained by analyzing the series of data obtained in the TEM images, as shown in Figure 3.

**Figure 4:**
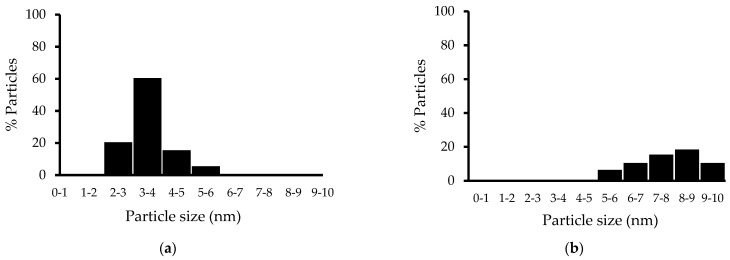
Size particle distribution of the Pt determinates by TEM for the photocatalyst Pt/TiO_2_-CeO_2_ 10 wt% (**a**) Sol-gel and (**b**) Impregnation.

Figure 4 shows how the particle size decreases, with the addition of CeO_2_ to the network of the support, that can suggest an increment of the specific surface area of the support. The micrographs reveal that the sizes of the metallic particles for the photocatalysts ranged from 2 to 6 nm. The smallest Pt particle size was observed in Pt/TiO_2_ CeO_2_ 10 wt%. 

### 3.5. X-ray Photoelectron Spectrometry (XPS)

**Figure 5:**
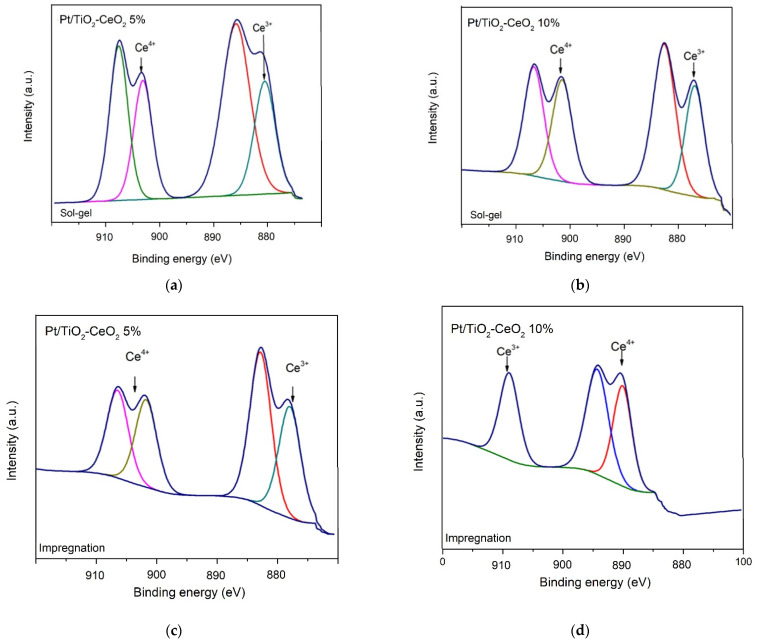
XPS spectra (Ce 3d region) for photocatalyst (**a**) Sol-gel Pt/TiO_2_-CeO_2_ 5%, (**b**) Sol-gel Pt/TiO_2_-CeO_2_ 10%, (**c**) Impregnation Pt/TiO_2_-CeO_2_ 5%, and (**d**) Impregnation Pt/TiO_2_-CeO_2_ 10%.

The binding energy and the atomic ratios of Pt, Ti and Ce, for the Pt/TiO_2_-CeO_2_ catalysts (5 and 10 wt%) prepared by impregnation and sol-gel are reported in Table 2. The relative abundance of the Pt^0−^ Pt^2+^ and Ce^3+^ Ce^4+^ species were calculated from the area under the curve of the respective peaks of the XPS spectra for the different catalysts (Figure 5). In Table 2, the corresponding binding energies for Pt 4f_(7/2)_ are shown; the values of the binding energies for the Pt/TiO_2_ and Pt/TiO_2_-CeO_2_ catalysts are around 73.0 to 75.9 eV corresponding to Pt^0^ and Pt^2+^. A shift in the binding energy towards higher energies can be observed with an increase the amount of cerium oxide in the Pt/TiO_2_-CeO_2_ catalysts (5% by weight and 10%). This is due mainly to the fact that CeO_2_ is considered as an oxygen supplier which makes the platinum species in the reduced state Pt^0^ transform to the oxidized species of Pt^2+^ [24,25,26].

**Figure 6:**
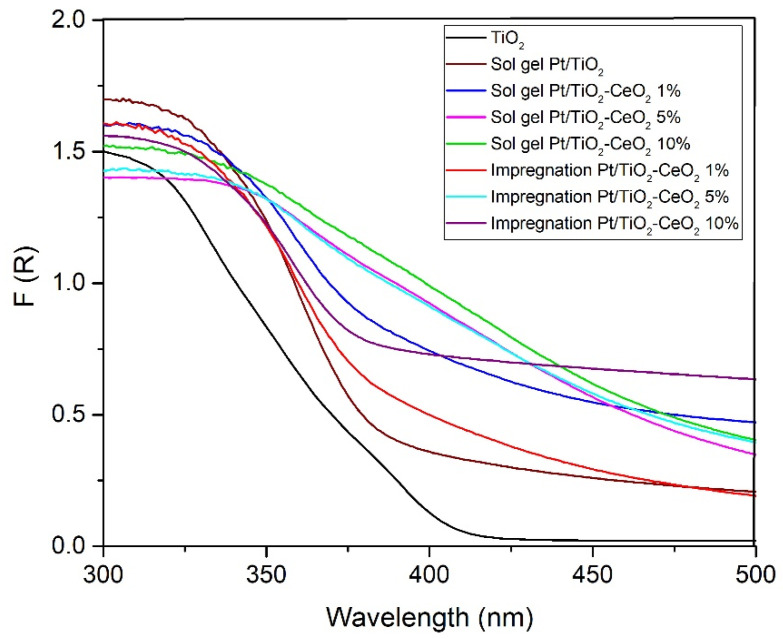
UV–vis spectra for the TiO_2_-CeO_2_ supports Sol-Gel and Impregnation.

Table 2 reports the binding energies for TiO_2_, which can have values ranging between 457.7–458.1 eV [27], as shown in Figure 6, which indicates that there was no modification due to the doping effect with the CeO_2_ content, nor with the preparation method of the Pt supports. The binding energy was also determined for the Ce 3d_5/2_ level (Table 2 and Figure 6); it was found in the region of 870–920 eV [28]. Relative abundance calculated from these XPS spectra showed that Ce^4+^ (oxidized) species increased with increasing CeO_2_ content relative to Ce^3+^ (reduced). Coinciding with Rocha et al. (2015), it is possible to observe that at a lower concentration of CeO_2_, a greater number of atoms in the Ce^3+^ oxidation state will be obtained.

### 3.6. UV–Vis for Band Gap

The photophysical properties UV–Vis absorption spectra of the catalysts were evaluated to investigate the effect of CeO_2_ on the support network. Figure 6 shows the spectra of UV–Vis materials by diffuse reflectance for sol-gel and impregnation methods. All samples have a shift between these wavelengths, which can be attributed to the transitions of the Ti-O electrons of the TiO_2_ and TiO_2_-CeO_2_ nanocrystals. 

Table 3 shows the results where a change in activation energy (3.02–2.8 eV) was observed for the TiO_2_-CeO_2_ samples from 1% to 5% by weight of CeO_2_, compared to the reference TiO_2_ in anatase phase (3.4 eV). The band gap energies were calculated by a linear fit of the slope to the abscissa and are reported in Table 3. It diminished from 3.45 eV, for the bare TiO_2_, to 2.82 eV, for the TiO_2_-CeO_2_ (at 5 wt%) sample. It is evident that cerium oxide modifies the bulk semiconductor properties of TiO_2_. The shift of the Eg band gap to a lower energy can be attributed to the incorporation of Ce^4+^ cations, which substitute some Ti^4+^ cations.

### 3.7. Photocatalysts Degradation of 2,4-Dichlorophenoxyacetic Acid

**Figure 7:**
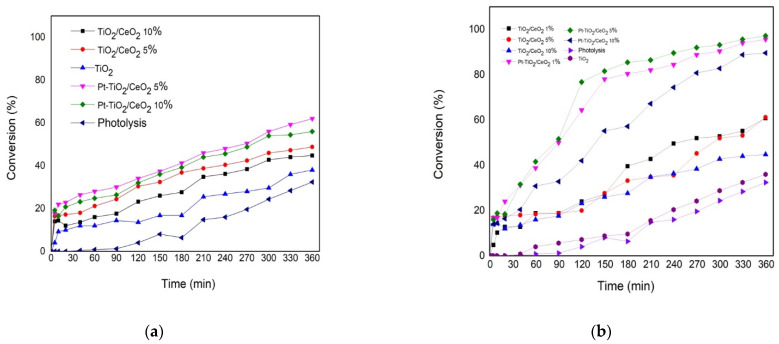
Photocatalysts degradation of 2,4-Dichlorophenoxyacetic acid. (**a**) Impregnation and (**b**) Sol-Gel materials.

The photocatalytic degradation reactions of 2,4-D acid were carried out at room temperature at 298 K for 6 h, with a concentration of 200 ppm of the reagent, followed by the UV absorption band of 283 that corresponds mainly to the transition electron n → π*, which is mainly attributed to the C-Cl bond [29,30]. The percentage conversion as a function of time for both supports and catalysts impregnated and prepared by the sol-gel method at 360 min of reaction are shown in Figure 7 and Table 4. 

The photocatalytic degradation of 2,4-D in the absence of support or catalyst had a conversion of 32% while the maximum conversion reached was 95% and 97% for the Pt/TiO_2_-CeO_2_ 1% and Pt/TiO_2_-CeO_2_ catalysts 5% prepared by the sol-gel method (Figure 7B), and the catalysts prepared by the impregnation method reached a maximum of 62% conversion. On the other hand, the supports prepared by impregnation reached a maximum of 49% TiO_2_ while those prepared by the sol-gel method reached up to 61% (Figure 7A). The highest yield achieved in the catalysts prepared by sol-gel Pt/TiO_2_-CeO_2_ 1% and Pt/TiO_2_-CeO_2_ 5% could be attributed to an optimal concentration of CeO_2_, which allows the insertion within the CeO_2_ of the TiO_2_ and leads to the deformation of the lattice, modifying the mobility of the oxygen atoms and favoring the oxidation-reduction process [26]. In contrast, the results of XPS in the Pt/TiO_2_-CeO_2_ 1% and Pt/TiO_2_-CeO_2_ 5% catalysts showed that the proportion of oxidized species of Pt ^2+^ and Ce ^4+^ are essential to function as oxygen scavengers, which are important in oxidation-reduction processes. Additionally, the smallest particle size in the catalysts prepared with the supports by sol-gel was in a range of 2 to 6 nm. This is due to a greater specific area due to a good integration of CeO_2_, which favors a better dispersion of the metallic nanoparticles on the surface of the supports, favoring the catalytic activity in the degradation of 2,4-D.

## 4. Conclusions

In the present work, the TiO_2_ and TiO_2_-CeO_2_ supports, prepared by the sol-gel method and increasing the CeO_2_ concentration in a 1–10 ratio in the TiO_2_ support network, significantly increased the pore diameter, affecting the specific surface area for the catalyst. On the other hand, in the supports prepared by impregnation, no important modification was observed, either in the area or in the pore diameter due to the addition of CeO_2_, since these remained constant. However, when comparing the results of both materials we can conclude that sol-gel supports can obtain pore diameters four times smaller than those obtained with impregnation. By having less exposed area, the Pt catalyst particles will be larger because they tend to agglomerate, as they do not have enough space to disperse efficiently. Affecting the catalytic activity of the material, the Pt particles, being well dispersed, favored the catalytic activity of the material. Another important fact is that it was possible to obtain Pt nanoparticles on the sol-gel supports in the order of 2 and 6 nm, dependent of the CeO_2_ content in the support. A cerium oxide shift in the energy band gap was observed in the Pt/TiO_2_-CeO_2_ photocatalysts. It is proposed that the high activity showed by the Pt/TiO_2_-CeO_2_ photo-catalysts can be due to a synergetic effect between the cerium oxide and the platinum of oxidizing agent.

## Figures and Tables

**Table 1:** Specific surface area, and pore diameter for TiO_2_-CeO_2_ catalysts support.

Support	Method	Diameter Pore (Å)	Specific Surface Area (m^2^/g)
TiO_2_	Sol-gel	52.54	185.59
TiO_2_-CeO_2_ 1%	Sol-gel	53.08	181.46
TiO_2_-CeO_2_ 10%	Sol-gel	77.51	104.36
TiO_2_-CeO_2_ 1%	Impregnation	295.06	43.56
TiO_2_-CeO_2_ 10%	Impregnation	304.05	43.61

**Table 2:** Binding energy and relative abundance of the different species obtained by XPS of the catalysts.

Support	Method	Binding Energy (eV)			Relative Abundance (%)		
		Pt (4f_7/2_)	Ti (2p_3/2_)	Ce (3d_5/2_)	Pt^0−^ Pt^2+^	Ti^4+^	Ce^3+^ Ce^4+^
Pt/TiO_2_	Sol-gel	73.0 75.9	458.1	-	80–20	100	-
Pt/TiO_2_-CeO_2_ 5%	Sol-gel	75.4 77.09–78.09	458	880–900	53–47	100	56–44
Pt/TiO_2_-CeO_2_ 10%	Sol-gel	75.9 77.09–78.09	458.1	881–900.1	47–53	100	52–47
Pt/TiO_2_-CeO_2_ 5%	Impregnation	-	457.7	880–900	-	100	48.8–51.19
Pt/TiO_2_-CeO_2_ 10%	Impregnation	-	465	880–900	-	100	38–62

**Table 3:** Band Gap Energy and Wavelengths.

Catalyst ID Name	Band Gap (eV)	Wavelengths (nm)
TiO_2_	3.45	359
Pt/TiO_2_	3.39	365
Pt/TiO_2_-CeO_2_- 1%	3.05	406
Pt/TiO_2_-CeO_2_- 5%	2.82	439

**Table 4:** Photocatalysts degradation of 2,4 Dichlorophenoxiacetyc acid.

Catalysts	Method	Pt (wt%)	X%	C_f_ (ppm)
TiO_2_	Commercial	-	38	160
TiO_2_-CeO_2_ 5%	Impregnation	-	49	128
TiO_2_-CeO_2_ 10%	Impregnation	-	45	138
Pt-TiO_2_-CeO_2_ 5%	Impregnation	1	62	95
Pt-TiO_2_-CeO_2_ 10%	Impregnation	1	56	110
TiO_2_	Sol-Gel	-	38	155
TiO_2_-CeO_2_ 1%	Sol-Gel	-	61	98
TiO_2_-CeO_2_ 5%	Sol-Gel	-	61	66
TiO_2_-CeO_2_ 10%	Sol-Gel	-	45	138
Pt-TiO_2_-CeO_2_ 1%	Sol-Gel	1	95	11
Pt-TiO_2_-CeO_2_ 5%	Sol-Gel	1	97	7
Pt-TiO_2_-CeO_2_ 10%	Sol-Gel	1	89	27
Photolysis	-	-	32	169

## Data Availability

Not applicable.
